# VEGF promotes endothelial progenitor cell differentiation and vascular repair through connexin 43

**DOI:** 10.1186/s13287-017-0684-1

**Published:** 2017-10-24

**Authors:** Lufeng Li, Huanyun Liu, Chunxin Xu, Mengyang Deng, Mingbao Song, Xuejun Yu, Shangcheng Xu, Xiaohui Zhao

**Affiliations:** 1 Institute of Cardiovascular Research, Xinqiao Hospital, Third Military Medical University, Chongqing, 400037 China; 2 Cardiovascular Department, First People’s Hospital of Chong Qing Liang Jiang New Zone, Chongqing, 401120 China; 3 Department of Occupational Health, Third Military Medical University, Chongqing, 400038 China

**Keywords:** Endothelial progenitor cell, Vascular endothelial growth factor, Connexin 43, Differentiation, Vascular repair

## Abstract

**Background:**

Endothelial progenitor cell (EPC) differentiation is considered crucial for vascular repair. Vascular endothelial growth factor (VEGF) induces EPC differentiation, but the underlying mechanism of this phenomenon remains unclear. Connexin 43 (Cx43) is reported to be involved in the regulation of stem cell differentiation. Therefore, we sought to determine whether Cx43 is involved in VEGF-induced EPC differentiation and vascular repair.

**Methods:**

Rat spleen-derived EPCs were cultured and treated with various concentrations of VEGF (0, 10, or 50 ng/mL), and the relationship between EPC differentiation and Cx43 expression was evaluated. Thereafter, fluorescence redistribution after photobleaching was performed to assess the relationship between adjacent EPC differentiation and Cx43-induced gap junction intercellular communication (GJIC). After carotid artery injury, EPCs pretreated with VEGF were injected into the tail veins, and the effects of Cx43 on vascular repair were evaluated.

**Results:**

EPCs cultured with VEGF exhibited accelerated differentiation and increased expression of Cx43. However, inhibition of Cx43 expression using short interfering RNA (siRNA) attenuated EPC GJIC and consequent EPC differentiation. VEGF-pretreated EPC transplantation promoted EPC homing and reendothelialization, and inhibited neointimal formation. These effects were attenuated by siRNA inhibition of Cx43.

**Conclusions:**

Our results from in vivo and in vitro experiments indicated that VEGF promotes EPC differentiation and vascular repair through Cx43.

**Electronic supplementary material:**

The online version of this article (doi:10.1186/s13287-017-0684-1) contains supplementary material, which is available to authorized users.

## Background

Vascular endothelial injury has been identified as a trigger of and critical contributor to atherosclerosis [1, 2]. Previous studies have suggested that endothelial progenitor cells (EPCs) derived from the bone marrow or spleen have the potential to be incorporated into injured areas and differentiate into endothelial cells (ECs), thereby contributing to the improvement of endothelial function [3, 4]. Vascular endothelial growth factor (VEGF) plays an important role in EPC differentiation and vascular repair [5, 6], which involves multiple signaling pathways including mitogen-activated protein kinase/extracellular signal-related kinase (MAPK/ERK) [7, 8]. However, to date, the mechanisms underlying VEGF-mediated EPC differentiation remain unclear.

Gap junctions (GJs), which are composed of connexins, are an important contributor to direct intercellular communications, mediating the exchange of ions, metabolites, and secondary messenger molecules between neighboring cells [9]. Among the 21 members of the connexin family, connexin 43 (Cx43) is reported to be a major GJ protein in multiple cells and is essential for the regulation of stem cell differentiation [10–12]. We found that blocking intercellular communication via Cx43 hemichannels reduces neointimal formation after vascular injury by inhibiting the proliferation and phenotypic modulation of smooth muscle cells [13]. Furthermore, it has been reported that blocking Cx43 expression inhibits GJ-mediated signal transfer between adjacent cells, thereby impairing EPC proliferation, migration, and angiogenesis; this can be visualized by observing the inhibition of fluorescence flow between cells [14, 15]. However, whether Cx43 participates in VEGF-induced EPC differentiation remains unclear. Therefore, in the present study, we investigated the effects of Cx43-mediated GJ function on EPC differentiation and vascular repair.

## Methods

### EPC culture and characterization

EPC culture and characterization were performed as previously described [16, 17]. Briefly, mononuclear cells (MNCs) were isolated from the spleens of Sprague-Dawley rats (male, 150–180 g; Chongqing, China) by density gradient centrifugation with Ficoll separation solution. After rinsing three times with phosphate-buffered saline (PBS; Sigma-Aldrich, St Louis, MO, USA), MNCs were seeded on gelatin-coated cell culture plates (1 × 10^6^ cells/cm^2^) and maintained in Dulbecco’s modified Eagle's medium (DMEM; Gibco, Waltham, MA, USA) supplemented with 20% fetal calf serum in a 5% CO_2_ incubator at 37 °C. The medium was changed every 3 days, and only attached cells were used in subsequent experiments.

For characterization, cells were incubated with 2.4 ng/mL DiI-labeled acetylated low-density lipoprotein (DiI-ac-LDL; Invitrogen, Carlsbad, CA, USA) for 1 h at 37 °C and fixed in 2% paraformaldehyde (PFA; CellChip Biotechnology, Shenzhen, China) for 15 min. After washing with PBS, cells were stained with 10 mg/mL fluorescein isothiocyanate-labeled *Ulex europaeus* agglutinin I (FITC-UEA-I; Sigma-Aldrich) for 1 h. The samples were rinsed three times with PBS and evaluated using laser scanning confocal microscopy (LSCM; Leica, Wetzlar, Germany). Cells positive for both DiI-ac-LDL and FITC-UEA-I were regarded as differentiating EPCs. Additionally, EPC phenotypes were determined by fluorescence-activated cell sorting (FACS) with two antibodies: FITC-conjugated anti-CD34 (Abcam, Cambridge, MA, USA) and phycoerythrin (PE)-conjugated anti-VEGF receptor 2 (VEGFR2; Abcam).

### Immunofluorescence analysis

To evaluate the expression of Cx43, EPCs were fixed in 4% PFA for 15 min and washed three times with PBS. Then, cells were permeabilized with 0.1% Triton X-100 for 15 min and treated with serum sealing fluid for 30 min. EPCs were first incubated with anti-Cx43 primary antibody (Abcam; 1:500), and then with FITC-labeled secondary antibody (Beyotime, Shanghai, China). After washing with PBS, EPCs were incubated with 4′,6-diamidino-2-phenylindole (DAPI) staining solution. Images were obtained using LSCM (Leica).

### Semi-quantitative reverse transcriptase (RT)-PCR

After incubation with VEGF (0, 10, or 50 ng/mL) for 24 h, EPCs were harvested. Total RNA was extracted with RNAiso Plus (Takara, Tokyo, Japan) according to the manufacturer’s instructions. DNA was obtained through RT-PCR using a PrimeScript RT reagent kit and Max PCR Master Mix (Takara) with total RNA as the template. The Cx43-specific primer (163 bp) was synthesized by Fulengen (Guangzhou, China). β-actin was used as a control, and the primer sequences (Takara) were 5′-CCGTAAAGACCTCTATGCCAAC-3′ (sense) and 5′-ACTCATCGTACTCCTGCTTGCT-3′ (antisense), for a product length of 227 bp.

### Western blot analysis

After incubation with VEGF (0, 10, or 50 ng/mL) for 24 h, EPCs were harvested. Cells were lysed with radioimmunoprecipitation assay (RIPA) buffer (Beyotime) containing the protease inhibitor phenylmethane sulfonyl fluoride (PMSF; 100:1). The lysates were centrifuged at 12,000 × *g* and 4 °C for 20 min, and the protein concentration of the supernatant was determined using the bicinchoninic acid assay kit (Beyotime). Identical concentrations of protein were subjected to SDS-PAGE and transferred to polyvinylidene fluoride (PVDF) membranes (EMD Millipore, Billerica, MA, USA). After treatment with 5% bovine serum albumin (BSA) blocking reagent (Solarbio, Beijing, China) for 60 min at 25 °C, membranes were further incubated with anti-Cx43, anti-CD31, anti-von Willebrand factor (vWF), anti-glyceraldehyde 3-phosphate dehydrogenase (GAPDH), or anti-β-actin (Abcam). After washing with PBS three times, membranes were probed with corresponding horseradish peroxidase-coupled secondary antibodies (Beyotime). Protein bands were visualized with an enhanced chemiluminescence (ECL) detection system (Pierce, Waltham, MA, USA) and quantified using a gel image analysis system (Bio-Rad, Hercules, CA, USA).

### RNA interference

Cells were transfected with 8 μl Cx43 short interfering RNA (siRNA; siCx43; SC6008, Santa Cruz Biotechnology, Dallas, TX, USA) for 6 h at 37 °C in a 5% CO_2_ incubator. Then, cells were incubated with DMEM containing 20% fetal calf serum for an additional 48 h. For this experiment, transfection reagent (SC29528, Santa Cruz Biotechnology), transfection medium (SC36868, Santa Cruz Biotechnology), and FITC-labeled control siRNA (SC37007, Santa Cruz Biotechnology) were used. The proteins generated were quantified via western blot.

### Fluorescence redistribution after photobleaching (FRAP)

FRAP experiments were performed as previously described [13, 18]. The control group was treated with saline for 24 h, and the experimental group was treated with 50 ng/mL VEGF for 24 h. The EPC + VEGF + siCx43 group was treated with 8 μl Cx43 siRNA for 6 h following VEGF treatment.

EPCs under different treatments were loaded with the Ca^2+^-insensitive dye 6-carboxy-fluorescein diacetate (10 μg/mL; Sigma-Aldrich) for 10 min at 37 °C. Then, FRAP results were measured using a confocal microscope (Leica). The dye was excited at 488 nm, and its emission recorded at 570 nm. Before bleaching, polygons were drawn around the cells chosen for bleaching, and three pre-bleached images were scanned with a weaker laser. The cells chosen for bleaching were then exposed to 50 scans with a laser at 95% intensity, and the recovery of fluorescence in the bleached cells was measured every 15 s over 3 min. After correction for background bleaching, the recovery of fluorescence in the bleached cells at 3 min was compared with that of the pre-bleached scans, and the recovery percentage was calculated.

### Differentiation assay

CD31 and vWF are two important surface antigens found on ECs. Therefore, we detected the expression of CD31 and vWF using a western blot to confirm the differentiation of EPCs into ECs.

### Carotid artery injury and identification

Rats were randomly assigned to several experimental groups with each group having three rats. The sham group received a carotid artery injury only. In the saline group, 100 μl saline was injected into the tail vein after carotid artery injury. In the EPC group, 2 × 10^6^ EPCs in 100 μl saline were injected after carotid artery injury. In the VEGF-pretreated EPC group, 2 × 10^6^ EPCs were pre-incubated with 50 ng/mL VEGF for 24 h and then injected into the tail vein after carotid artery injury. In the VEGF-pretreated EPC + siCx43 group, 2 × 10^6^ EPCs were incubated with 8 μl siCx43 for 6 h and 50 ng/mL VEGF for 24 h and then injected into the tail vein after carotid artery injury.

Carotid artery injury was performed as previously described [19]. Briefly, rats were anesthetized with pentobarbital sodium at a dose of 40 mg/kg. The left carotid artery was exposed through a midline incision in the neck. Several silk sutures were applied to temporarily restrict blood flow to surgical areas. Then, a 1.5-F Fogarty balloon catheter (Baxter, Deerfield, IL, USA) was inserted through the external carotid artery, inflated, and passed three times along the segment. After the external carotid artery was permanently ligated, blood flow to the internal carotid artery was restored. The skin was closed with a single suture using 6-0 silk. Carotid arteries were harvested 7 or 28 days after carotid artery injury. Intimal hyperplasia was observed through hematoxylin and eosin (H&E; Solarbio) staining using an inverted microscope.

### EPC differentiation after transplantation

Before transplantation, EPCs (2 × 10^6^) were incubated with 2.4 g/mL DiI-ac-LDL for 1 h. Then, the cells were washed with PBS and injected into the rat tail vein in 100 μl saline after carotid artery injury. Carotid arteries were harvested 3 days after injury. After perfusion fixation with 4% PFA, the vessels were washed with PBS and embedded in optimal cutting temperature (OCT; Sakura, Torrance, CA, USA) compound. Subsequently, frozen sections of the carotid artery were prepared and incubated with FITC-UEA-I.

### Assessment of reendothelialization

Assessment of reendothelialization was performed as previously described [17]. One week after carotid artery injury, 200 μl 5% Evans blue (Sigma-Aldrich) was injected into the heart. Injured vessels were harvested 15 min later and then fixed in 4% PFA (Sigma-Aldrich). The reendothelialized area was defined as the area that was not stained with Evans blue dye. Morphometric analysis of the reendothelialization rate was performed using Image-Pro Plus 5.1 (Media Cybernetics Inc., Rockville, MD, USA).

### Assessment of intimal hyperplasia

Assessment of intimal hyperplasia was conducted as previously described [17]. Two weeks after carotid artery injury, injured vessels were harvested. The vessels were fixed in 4% PFA and embedded in OCT compound; thereafter, carotid cross-sections were prepared for H&E staining. All sections were examined under an inverted microscope (Leica). Morphometric analysis of the intimal/medial area was performed using Image-Pro Plus 5.1 (Media Cybernetics Inc.).

### Statistical analysis

All values are expressed as the mean ± standard deviation (SD). Comparisons between multiple groups were tested by one-way analysis of variance (ANOVA) followed by Fisher’s least significant difference (LSD) test. Statistical analyses were performed using SPSS v19.0 (IBM, Armonk, NY, USA). A value of *p* < 0.05 was considered statistically significant.

## Results

### Characterization of spleen-derived EPCs

**Fig. 1:**
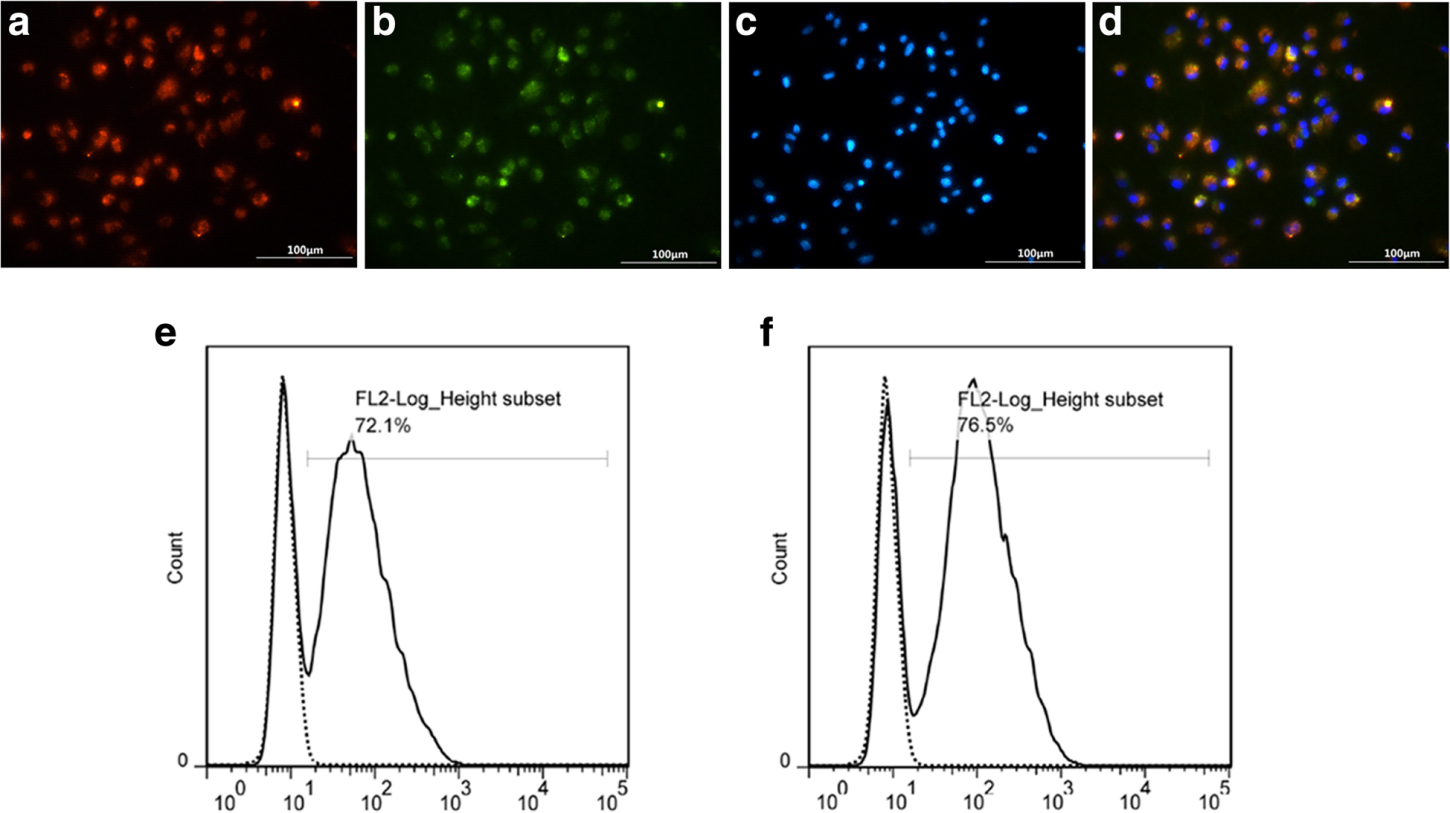
Spleen-derived mononuclear cells (MNCs) differentiate into cells with the characteristics of endothelial progenitor cells (EPCs) in vitro. **a**–**d** Cells double-labeled for DiI-labeled acetylated low-density lipoprotein (DiI-ac-LDL; *red*) and fluorescein isothiocyanate (FITC)-lectin binding (*green*) were identified as EPCs (*yellow*). Cell nuclei were stained with DAPI (*blue*). **e**, **f** Adherent MNCs were analyzed for expression of CD34 and vascular endothelial growth factor receptor 2 (VEGFR2), respectively, by fluorescence-activated cell sorting (FACS). *Dotted histograms* represent isotype controls. Representative images from at least three experiments are shown. Scale bar = 100 μm

Isolated spleen-derived MNCs exhibited a spindle-shaped morphology after 4–7 days of culture (see Additional file 1). Most cells (92.00 ± 2.23%) were positive for both DiI-ac-LDL and FITC-UEA-I and were therefore identified as EPCs (Fig. 1a–d). Furthermore, the percentages of cells positive for CD34 (stem cell marker) and VEGFR2 (EC marker) were 67.77 ± 3.84% and 73.80 ± 2.65%, respectively (n = 3; Fig. 1e and f).

### VEGF promotes the expression of Cx43 in EPCs

**Fig. 2:**
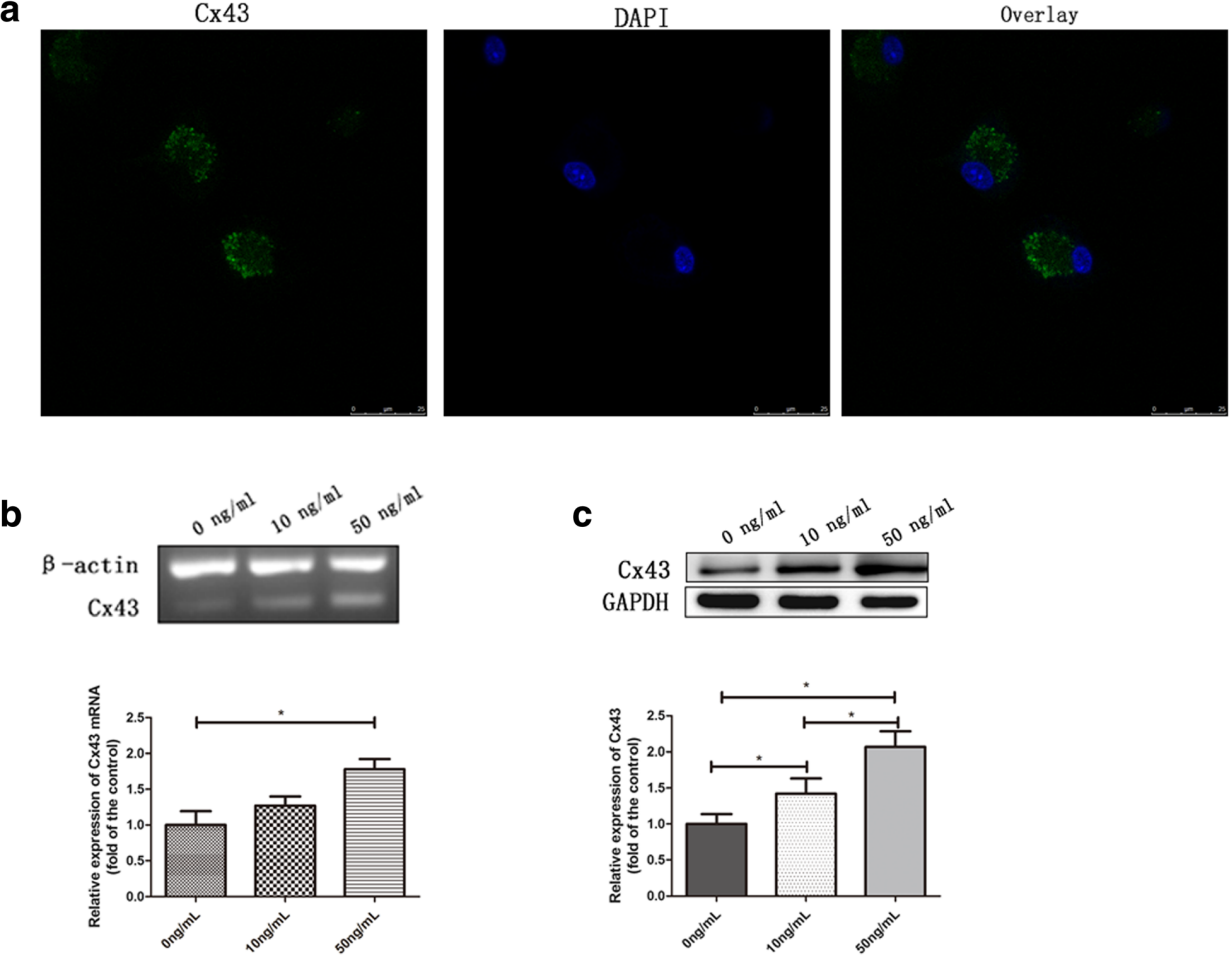
Vascular endothelial growth factor (VEGF) promotes expression of connexin 43 (Cx43) in EPCs. **a** Cx43 was detected in the cytomembrane and cytoplasm of EPCs. Scale bar = 25 μm. **b** *Cx43* mRNA expression significantly increased after VEGF treatment (n = 3). **c** VEGF enhanced the protein expression levels of Cx43 (n = 5). *p < 0.05

Immunofluorescence analysis indicated that Cx43 was located in both the cytomembrane and cytoplasm of EPCs (green in Fig. 2a). Control cells without primary antibody showed no staining (see Additional file 2). With increasing concentrations of VEGF, we observed a clear increase in relative *Cx43* mRNA expression in EPCs (1.78 ± 0.25 vs. 1.00 ± 0.33 with 50 and 0 ng/mL VEGF, respectively; *p* < 0.05; Fig. 2b). In addition, VEGF significantly augmented relative Cx43 protein expression in EPCs (2.07 ± 0.49 vs. 1.42 ± 0.47 vs. 1.00 ± 0.31 with 50, 10, and 0 ng/mL VEGF, respectively; *p* < 0.05; Fig. 2c).

### Transfection efficiency of Cx43 siRNA

Western blot analysis was conducted to verify the transfection efficiency of Cx43 siRNA. After transfection for 48 h, levels of Cx43 protein in the siCx43 group were considerably decreased compared to the negative control group (see Additional file 3).

### VEGF promotes Cx43-mediated GJ intercellular communication between adjacent EPCs

**Fig. 3:**
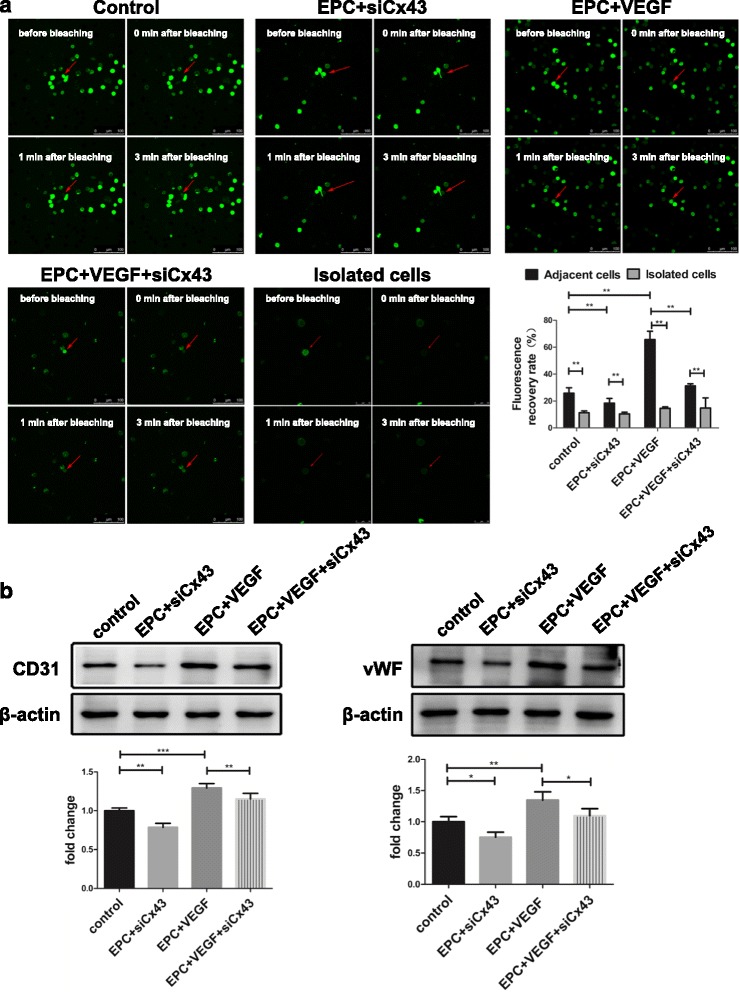
The role of Cx43-mediated gap junction intercellular communication (GJIC) in VEGF-induced EPC differentiation. **a** VEGF induced a significant increase in GJIC, while the silencing of Cx43 decreased GJIC (n = 6). The *red arrow* represents bleached cells. Scale bar = 100 μm. **b** VEGF promoted the expression of CD31 and von Willebrand factor (vWF). However, the silencing of Cx43 reduced the expression of CD31 and vWF (n = 3). *p < 0.05; **p < 0.01; ***p < 0.001

FRAP was used to detect functional GJ intercellular communication (GJIC) between adjacent EPCs. The fluorescence recovery rate was higher in adjacent EPCs than in isolated EPCs, with significant differences between adjacent and isolated cells in the control group (25.85 ± 4.00% vs. 11.33 ± 1.39%; *p* < 0.01), adjacent and isolated cells in the EPC + siCx43 group (18.52 ± 3.38% vs. 10.43 ± 1.23%; *p* < 0.01), adjacent and isolated cells in the EPC + VEGF group (65.65 ± 6.24% vs. 14.57 ± 1.16%; *p* < 0.01), and adjacent and isolated cells in the EPC + VEGF + siCx43 group (31.31 ± 1.53% vs. 14.83 ± 7.48%; *p* < 0.01). Transfection with Cx43 siRNA decreased the fluorescence recovery of bleached cells (18.52 ± 3.38% vs. 25.85 ± 4.00% in the EPC + siCx43 and control groups, respectively; *p* < 0.01). Administration of VEGF increased the fluorescence recovery of bleached cells (65.65 ± 6.24% vs. 25.85 ± 4.00% in the EPC + VEGF and control groups, respectively; *p* < 0.01), whereas pretreatment with Cx43 siRNA reduced fluorescence recovery (31.31 ± 1.53% vs. 65.65 ± 6.24% in the EPC + VEGF + siCx43 and EPC + VEGF groups, respectively; *p* < 0.01; Fig. 3a).

### VEGF promotes Cx43-mediated differentiation of EPCs

The differentiation of EPCs is characterized by the expression of EC markers (CD31 and vWF). To assess the effect of VEGF on Cx43-mediated EPC differentiation, EPCs were cultured with VEGF and/or Cx43 siRNA. Transfection with Cx43 siRNA blocked the relative protein expression of CD31 (0.79 ± 0.05 vs. 1.00 ± 0.03 in the EPC + siCx43 and control groups, respectively; *p* < 0.01) and vWF (0.75 ± 0.08 vs. 1.00 ± 0.08 in the EPC + siCx43 and control groups, respectively; *p* < 0.05). EPCs cultured with VEGF displayed higher relative protein expression of CD31 (1.29 ± 0.05 vs. 1.00 ± 0.03; *p* < 0.001) and vWF (1.35 ± 0.13 vs. 1.00 ± 0.08; *p* < 0.01) than control EPCs. However, silencing of Cx43 attenuated the relative protein expression of CD31 (1.15 ± 0.07 vs. 1.29 ± 0.05; *p* < 0.01) and vWF (1.09 ± 0.11 vs. 1.35 ± 0.13; *p* < 0.05) in EPCs treated with VEGF (Fig. 3b).

### VEGF pretreatment increases EPC homing via Cx43

**Fig. 4:**
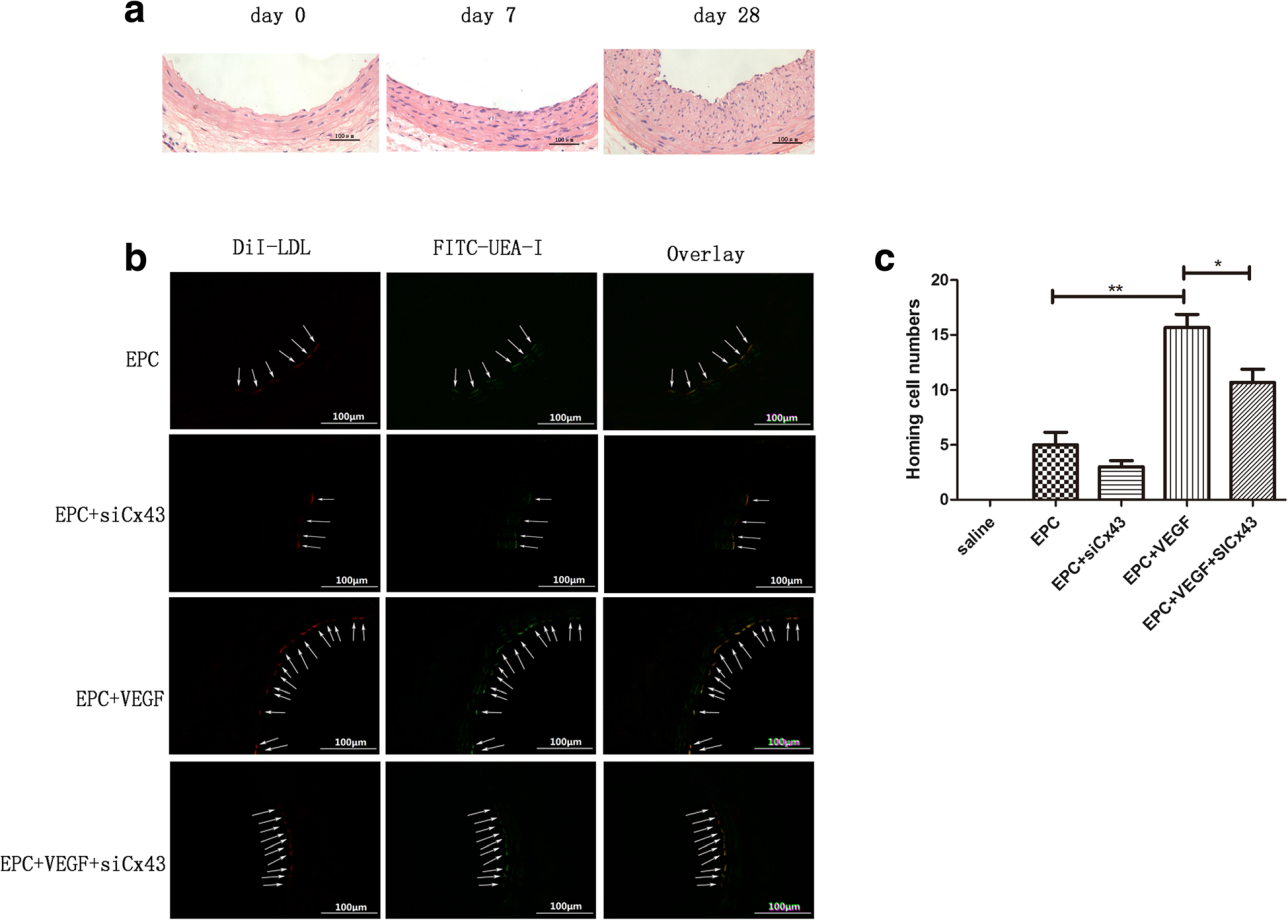
VEGF pretreatment increases EPC homing via Cx43. **a** Significant neointimal formation was detected on days 7 and 28 after injury. Scale bar = 100 μm. **b** VEGF pretreatment promoted the homing of EPCs. However, the silencing of Cx43 attenuated EPC homing. Scale bar = 100 μm. *White arrows* represent homing EPCs. **c** Quantitative analysis of EPC homing (n = 3). *p < 0.05; **p < 0.01

H&E staining showed obvious neointima on days 7 and 28, confirming carotid artery injury (Fig. 4a). To determine whether EPCs were incorporated into the injured vessel walls and differentiated into ECs, animals received 2 × 10^6^ DiI-labeled EPCs via tail vein injection. DiI-labeled EPCs, identified as red fluorescent cells, were seen lining the lumen via co-staining for the endothelial marker FITC-UEA. No DiI-labeled cells were observed in the saline group (see Additional file 4). Although differences were found between the EPC + siCx43 and EPC groups (3 ± 1 vs. 5.00 ± 2.00), this difference was not statistically significant (*p* = 0.22). There was a significant increase in the number of EPCs in the injured areas after VEGF pretreatment (15.67 ± 2.08 vs. 5.00 ± 2.00 in the EPC + VEGF and EPC groups, respectively; *p* < 0.01), which was significantly inhibited by Cx43 interference (10.67 ± 2.08 vs. 15.67 ± 2.08 in the EPC + VEGF + siCx43 and EPC + VEGF groups, respectively; *p* < 0.05; Fig. 4b and c).

### VEGF pretreatment before EPC transplantation promotes reendothelialization via Cx43

**Fig. 5:**
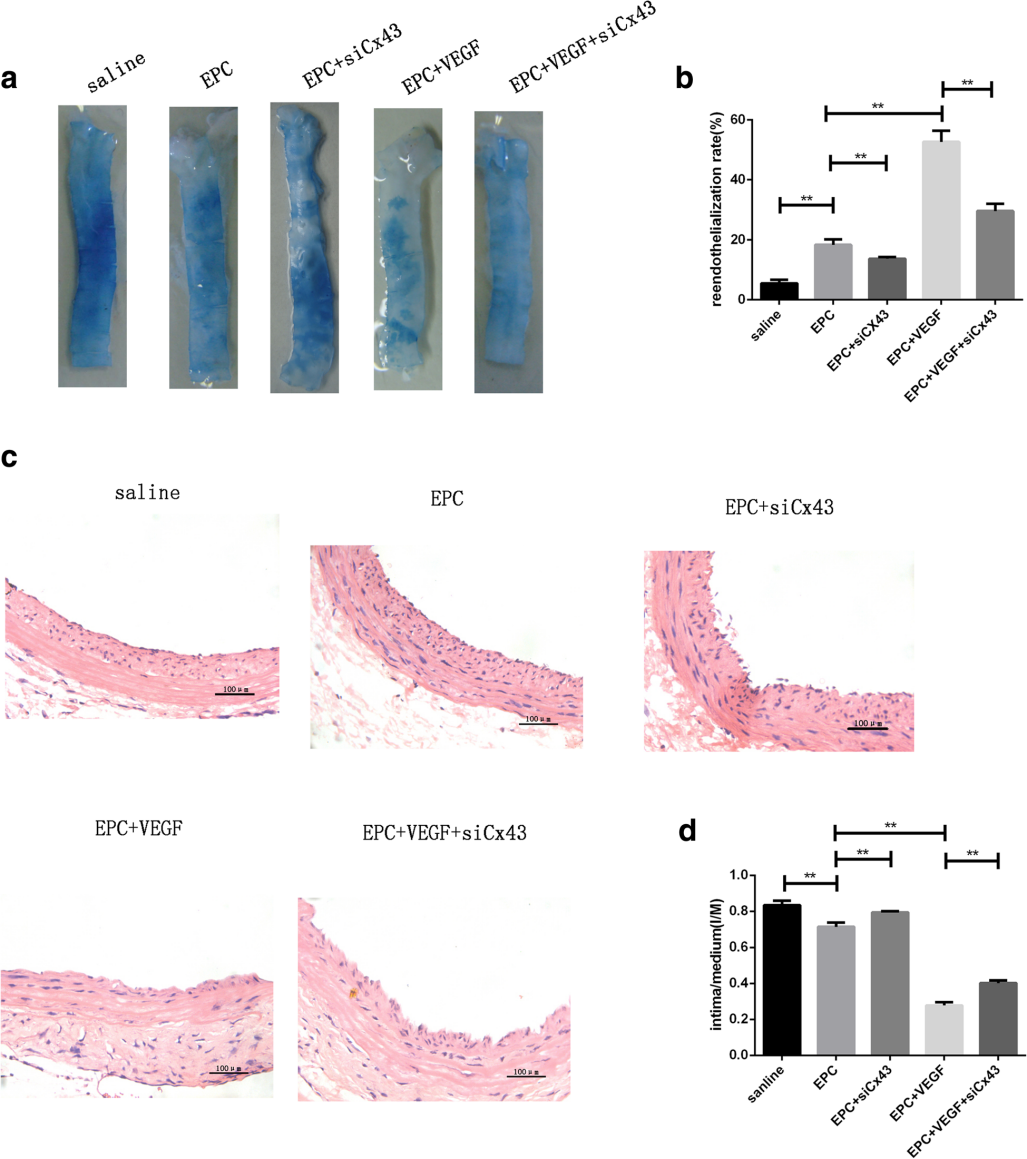
VEGF pretreatment before EPC transplantation promotes reendothelialization and inhibits neointimal formation through Cx43. **a** EPC transplantation promoted reendothelialization, which was further enhanced by VEGF stimulation. However, the silencing of Cx43 attenuated neointimal formation. **b** Quantitative analysis of reendothelialization (n = 3). **c** EPC transplantation inhibited neointimal proliferation, which was further enhanced by VEGF pretreatment. However, the silencing of Cx43 increased neointimal formation compared to the EPC + VEGF group. Scale bar = 100 μm. **d** Quantitative analysis of intimal proliferation (n = 3). **p < 0.01

To determine whether VEGF promoted endothelium recovery via Cx43, rats were injected with Evans blue 7 days after carotid artery injury. Reendothelialization was represented by the ratio of the reendothelialized area (white) to the total area (white + blue). Reendothelialization significantly increased after EPC transplantation (18.39 ± 1.83% vs. 5.51 ± 1.18% in the EPC and saline groups, respectively; *p* < 0.01). Transfection with Cx43 siRNA inhibited reendothelialization (13.73 ± 0.575% vs. 18.39 ± 1.83% in the EPC + siCx43 and EPC groups, respectively; *p* < 0.01). This effect was strengthened by VEGF pretreatment (52.66 ± 3.78% vs. 18.39 ± 1.83% in the EPC + VEGF and EPC groups, respectively; *p* < 0.01). However, the silencing of Cx43 attenuated reendothelialization (29.58 ± 2.44% vs. 52.66 ± 3.78% in the EPC + VEGF + siCx43 and EPC + VEGF groups, respectively; *p* < 0.01; Fig. 5a and b).

### VEGF pretreatment before EPC transplantation inhibits neointima formation via Cx43

The transplantation of EPCs significantly inhibited neointimal development in injured vessels, as determined by the ratio of the intimal to medial areas (0.714 ± 0.024 vs. 0.835 ± 0.025 in the EPC and saline groups, respectively; *p* < 0.01). Transfection with Cx43 siRNA impaired the ability of EPC inhibition of intimal proliferation (0.794 ± 0.065 vs. 0.714 ± 0.024 in the EPC + siCx43 and EPC groups, respectively; *p* < 0.01). VEGF pretreatment further inhibit neointima formation (0.278 ± 0.018 vs. 0.714 ± 0.024 in the EPC + VEGF and EPC groups, respectively; *p* < 0.01). However, Cx43 silencing increased intimal hyperplasia (0.402 ± 0.116 vs. 0.278 ± 0.018 in the EPC + VEGF + siCx43 and EPC + VEGF groups, respectively; *p* < 0.01; Fig. 5c and d).

## Discussion

Our results from in vivo and in vitro experiments indicated that VEGF promotes EPC differentiation and vascular repair through Cx43.

### EPCs promotes endothelial and vascular repair

The endothelium serves as a barrier between vessel wall and blood, and plays an essential role in regulating vascular homeostasis. Promoting endothelial generation is a therapeutic option for vascular repair; it has been demonstrated that EPCs—derived from hematopoietic stem cells—can mobilize into peripheral blood, home to an injured zone, differentiate into ECs, and promote vascular repair [4, 20].

### VEGF is a crucial regulator in vascular injury repair

VEGF has been shown to induce angiogenesis, accelerating endothelial repair and preventing intimal hyperplasia after vascular injury in vitro and in vivo [21–24]. One study found that EPCs transfected with adenovirus encoding for VEGF are more capable of homing to denuded areas and differentiating into ECs [25]. In our study, we also found that VEGF promotes EPC differentiation, homing, and repair of injured blood vessels.

### Mechanism of VEGF-induced EPC differentiation is unclear

It has been reported that VEGF induces EPC differentiation in in vivo and in vitro experiments, and therefore contributes to vascular repair [25–27]. Several mechanisms are suggested to be involved in this process. Some reports indicate that VEGF appears to regulate EPC differentiation through PI3K/Akt/eNOS signaling [28, 29]; one report suggests that the Akt/HDAC3/p53 pathway is involved [30]; and the serine/threonine-protein kinase Pim-1 [7, 27], as well as the MAPK/ERK signaling pathway [8, 31], also appear to participate. However, the specific mechanisms of VEGF-induced EPC differentiation and blood vessel repair remain unclear.

### Cx43 potentially modulates EPC differentiation

Of the 21 known connexins, vascular endothelial cells primarily express three: Cx37, Cx40, and Cx43 [18], all of which facilitate the propagation of electrical and chemical signals along the vessel wall. GJs in EPCs mainly consist of Cx43, and EPCs treated with Cx43 siRNA lose their therapeutic potential [14]. These findings link the adjustment of Cx43-mediated EPC function to the initiation and promotion of atherosclerosis. There is accumulating evidence that Cx43 is capable of inducing GJ formation between stem cells or between stem cells and mature differentiated cells, and that downregulation or upregulation of Cx43 can suppress or boost stem cell differentiation, respectively [11, 12, 32, 33]. It has also been demonstrated that Cx43-mediated GJs participate in EPC proliferation, migration, and angiogenesis [14]. Therefore, we aimed to directly test whether CX43 participates in EPC differentiation. Our study shows that VEGF promotes EPC differentiation and vascular repair through Cx43-mediated GJs.

### Possible mechanisms for Cx43 induction of EPC differentiation

Regarding the underlying mechanisms by which Cx43 induces EPC differentiation, cAMP/PKA and IP3/Ca2+ are proposed to be involved. One study found that cyclic adenosine monophosphate (cAMP) enhanced VEGF-induced EPC differentiation through activation of protein kinase A (PKA) signaling, and that blockade or upregulation of PKA expression reduced or enhanced the effect, respectively [34, 35]. These findings demonstrate that GJ-mediated exchange of cAMP could modulate EPC differentiation through PKA signaling.

Ca^2+^ is an important second messenger in the regulation of cell physiological functions; and inositol triphosphate 3 (IP3) may open calcium channels in the endoplasmic reticulum and increase intracellular calcium concentration. The distribution and transfer of Ca^2+^ through GJs may activate specific proteins, or may combine with specific enzymes, modulating cellular functions. Some studies have suggested that VEGF may activate phospholipase C (PLC-γ), hydrolyze phosphatidylinositol-4,5-bisphosphate (PIP2), increase cellular calcium concentration, regulate the expression of endothelial differentiation-related genes, and promote EPC differentiation [36, 37]. Therefore, the Cx43-mediated exchange of intercellular second messengers such as Ca^2+^ is clearly a potential mechanism by which Cx43 regulates EPC differentiation.

### Exploration of potential therapeutic applications

Recent efforts have been made to apply the differentiation-promoting capabilities of VEGF and Cx43 to clinical research settings. Several studies report that EPCs captured on VEGF-bound stents display rapid differentiation [38, 39], and Cx43 has been demonstrated to have an important role in maintaining the normal physiological functions of EPCs and ECs [14, 40, 41]. The injection of targeting nanoparticles loaded with Cx43-overexpressing adenoviruses, combined with EPCs, might be an effective method of treating vascular injuries and vascular pathologies. Alternatively, using stents to load Cx43-overexpressing EPCs into blood vessels is another possible therapeutic treatment for vascular injuries and diseases.

### Limitations

We believe that cell density plays a role in Cx43-mediated, VEGF-induced EPC differentiation. Our decisions to use cell densities of 1 × 10^6^ cells/cm^2^ for our in vitro experiments and 2 × 10^6^ cells/100 μL for our in vivo experiments were based on methods described in previous studies [19], but we hope to elucidate the specific influence of cell density in the future. Although we were able to demonstrate that VEGF promoted EPC differentiation through Cx43-induced GJs, we were unable to explore additional mechanisms of EPC differentiation because of limited experimental conditions. We hope to conduct future experiments that will employ microfabrication and laser-guided cell micropatterning techniques [33], as well as dual patch clamp recording techniques [42].

## Conclusions

In summary, we report that VEGF, at least in part, promotes EPC differentiation and vascular repair via Cx43. Our findings indicate that Cx43 is a potential therapeutic target for blood vessel injury diseases.

## Additional files

Additional file 1: Morphology of mononuclear cells (MNCs). (A) Smaller cells were diffusely distributed in culture solution after 1 day. (B) Cells became larger after culture for 4 days. (C) Cells exhibited a spindle-shaped, endothelial cell-like morphology after culture for 7 days. (TIF 1954 kb)

Additional file 2: Nonspecific fluorescence detection of endothelial progenitor cells (EPCs). EPCs were incubated with saline instead of anti-Cx43 primary antibody, and nuclei were stained with DAPI. Scale bar = 25 μm. (TIF 736 kb)

Additional file 3: Efficiency of connexin 43 (Cx43) short interfering RNA (siRNA). Treatment with Cx43 siRNA effectively reduced Cx43 protein expression (n = 3). *p < 0.05. *NC* negative control. (TIF 242 kb)

Additional file 4: Fluorescent tracer technique employed to detect saline group homing. No EPCs were observed in the area of the injured vessel. (TIF 2403 kb)

## Abbreviations

ANOVA: Analysis of variance
BSA: Bovine serum albumin
Cx43: connexin 43
DAPI: 4′,6-diamidino-2-phenylindole
DiI-ac-LDL: DiI-labeled acetylated low-density lipoprotein
DMEM: Dulbecco’s modified Eagle’s medium
EC: Endothelial cell
ECL: Enhanced chemiluminescence
EPC: Endothelial progenitor cell
FACS: Fluorescence-activated cell sorting
FITC-UEA-I: Fluorescein isothiocyanate (FITC)-labeled *Ulex europaeus* agglutinin I
FRAP: Fluorescence redistribution after photobleaching
GAPDH: Glyceraldehyde-3-phosphate dehydrogenase
GJ: Gap junction
GJIC: Gap junction intercellular communication
H&E: Hematoxylin and eosin
LSCM: Laser scanning confocal microscopy
LSD: Least significant difference
MAPK/ERK: Mitogen-activated protein kinase/extracellular signal-related kinase
MNC: Mononuclear cell
OCT: Optimal cutting temperature
PBS: Phosphate-buffered saline
PE: Phycoerythrin
PFA: Paraformaldehyde
PKA: Protein kinase A
PMSF: Phenylmethane sulfonyl fluoride
PVDF: Polyvinylidene fluoride
RIPA: Radioimmunoprecipitation assay
RT: Reverse transcriptase
SD: Standard deviation
siRNA: Short interfering RNA
VEGF: Vascular endothelial growth factor
VEGFR: Vascular endothelial growth factor receptor
vWF: von Willebrand factor
