# Supplementary figures and images for: VEGF promotes endothelial progenitor cell differentiation and vascular repair through connexin 43
Source: Stem Cell Res Ther. 2017 Oct 24;8:237. doi: 10.1186/s13287-017-0684-1 (PMC5655878; doi:10.1186/s13287-017-0684-1)

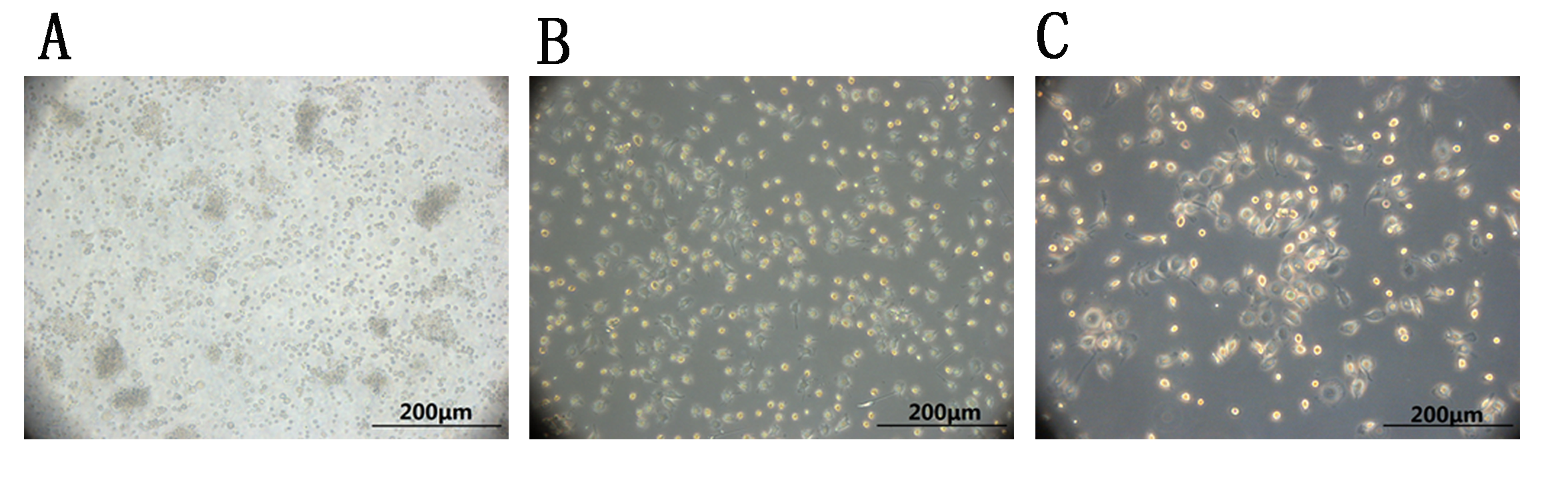

Supplement: Supplementary file 1 — Morphology of mononuclear cells (MNCs). (A) Smaller cells were diffusely distributed in culture solution after 1 day. (B) Cells became larger after culture for 4 days. (C) Cells exhibited a spindle-shaped, endothelial cell-like morphology after culture for 7 days. (TIF 1954 kb) [file 13287_2017_684_MOESM1_ESM.tif]

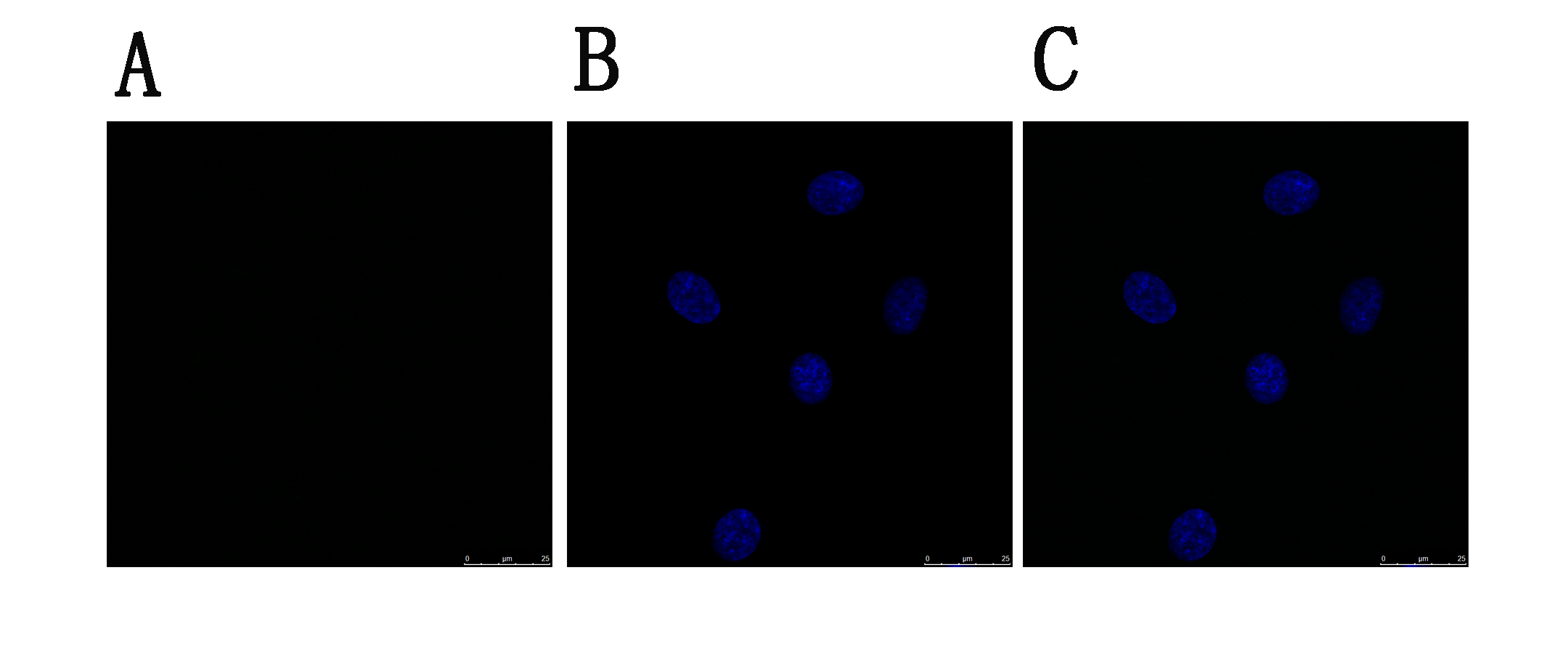

Supplement: Supplementary file 2 — Nonspecific fluorescence detection of endothelial progenitor cells (EPCs). EPCs were incubated with saline instead of anti-Cx43 primary antibody, and nuclei were stained with DAPI. Scale bar = 25 μm. (TIF 736 kb) [file 13287_2017_684_MOESM2_ESM.tif]

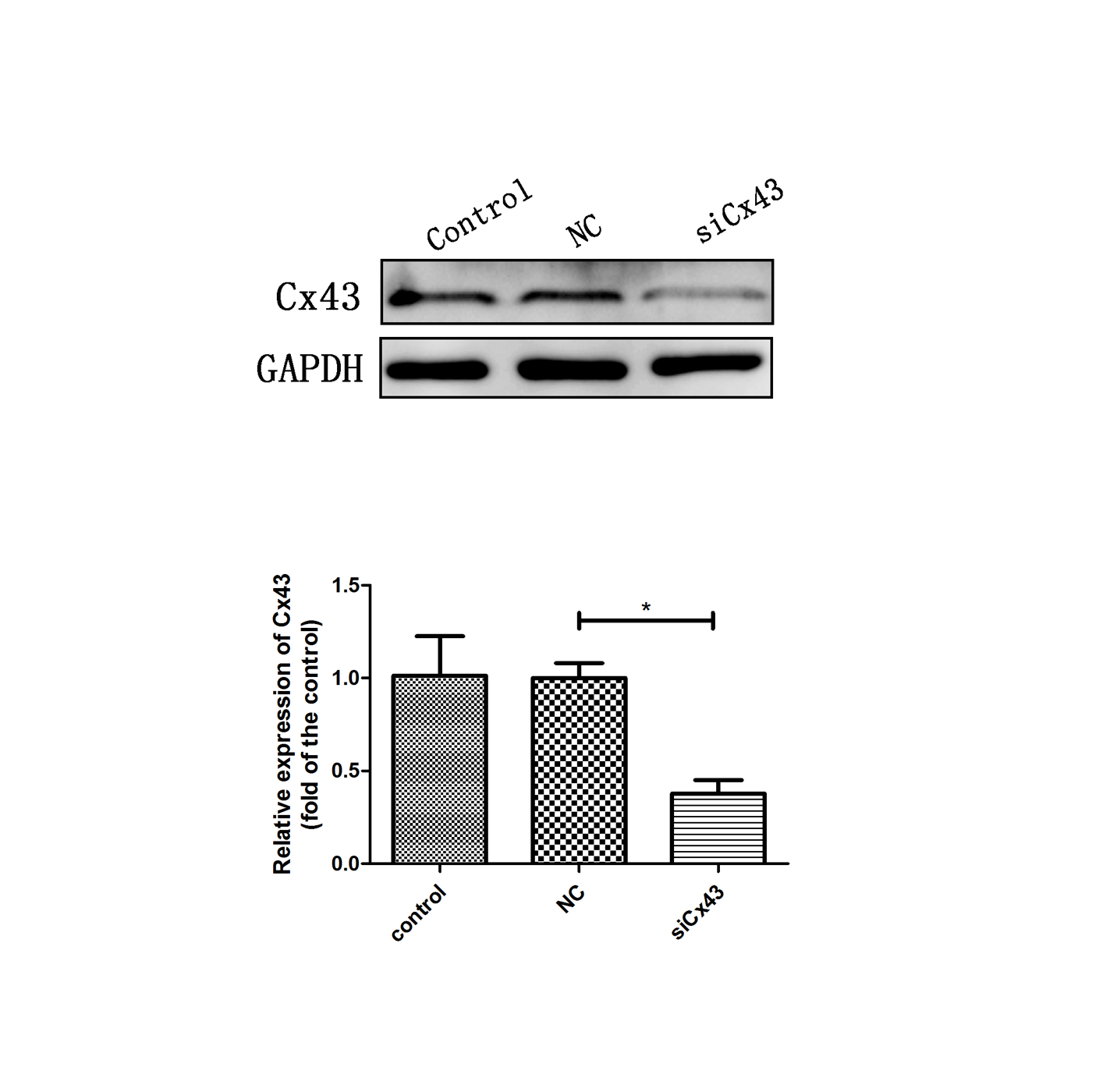

Supplement: Supplementary file 3 — Efficiency of connexin 43 (Cx43) short interfering RNA (siRNA). Treatment with Cx43 siRNA effectively reduced Cx43 protein expression (n = 3). *p < 0.05. NC negative control. (TIF 242 kb) [file 13287_2017_684_MOESM3_ESM.tif]

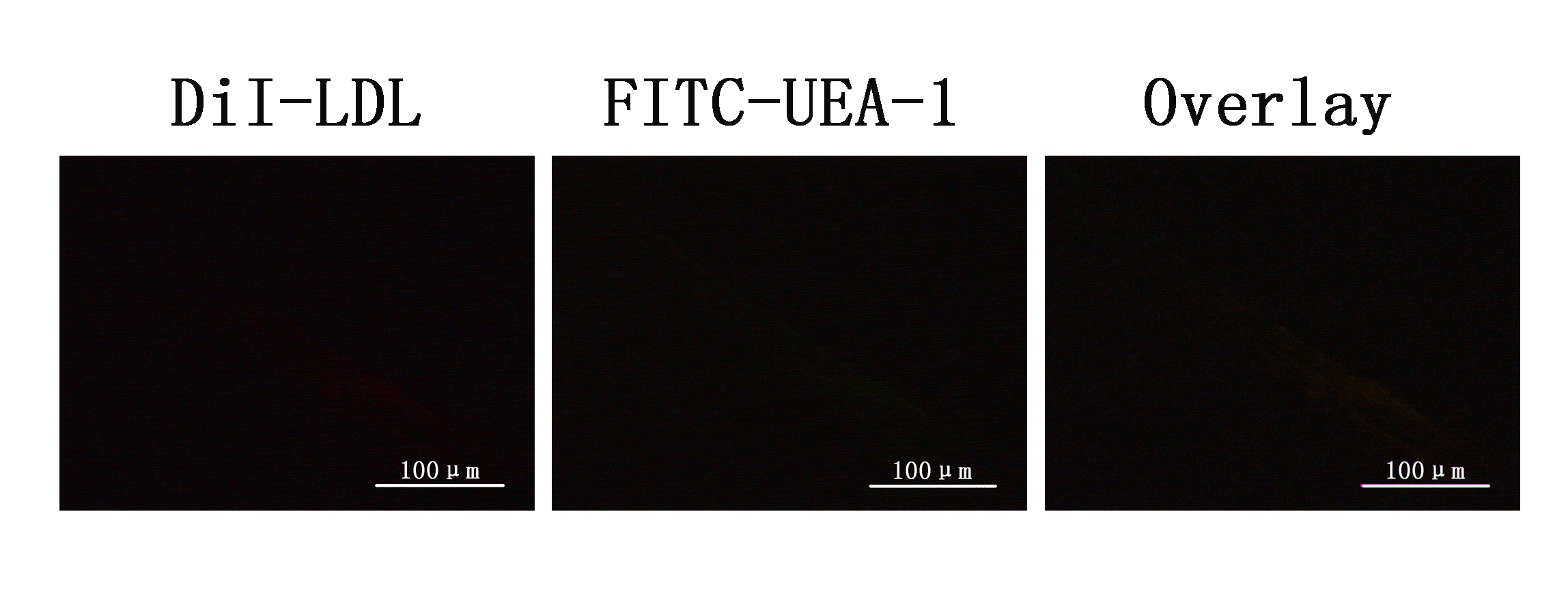

Supplement: Supplementary file 4 — Fluorescent tracer technique employed to detect saline group homing. No EPCs were observed in the area of the injured vessel. (TIF 2403 kb) [file 13287_2017_684_MOESM4_ESM.tif]
